# Editorial: Impact of Traumatic Brain Injuries on Participation in Daily Life and Work: Recent Research and Future Directions

**DOI:** 10.3389/fneur.2019.01153

**Published:** 2019-11-01

**Authors:** Nada Andelic, Marianne Løvstad, Anne Norup, Jennie Ponsford, Cecilie Røe

**Affiliations:** ^1^Department of Physical Medicine and Rehabilitation, Oslo University Hospital, Oslo, Norway; ^2^Research Centre for Habilitation and Rehabilitation Models & Services (CHARM), Faculty of Medicine, University of Oslo, Oslo, Norway; ^3^Research Department, Sunnaas Rehabilitation Hospital, Bjørnemyr, Norway; ^4^Department of Psychology, Faculty of Social Sciences, University of Oslo, Oslo, Norway; ^5^Traumatic Brain Injury Unit, Department of Neurorehabilitation, Rigshospitalet, Copenhagen, Denmark; ^6^Department of Psychology, Faculty of Health Sciences, University of Southern Denmark, Odense, Denmark; ^7^Monash Epworth Rehabilitation Research Centre, Epworth Healthcare, Melbourne, VIC, Australia; ^8^Turner Institute for Brain and Mental Health, School of Psychological Sciences, Monash University, Melbourne, VIC, Australia

**Keywords:** brain injury, functioning, disability, long-term outcomes, rehabilitation, healthcare services

A large proportion of individuals with traumatic brain injuries (TBI) sustain long-term physical, cognitive, and emotional impairments that have a profound impact on their everyday level of functioning, community participation, and reintegration (1). Participation in daily life activities and work is identified as one of the most important outcomes of TBI-rehabilitation by patients, their families and healthcare professionals. Identifying predictors for long-term participation is complicated, as there is a complex interaction between several influential factors (2). For example, motor and cognitive deficits appear to have significant impact on participation in the early stages, whereas personal, emotional and social factors play a major role in later stages of TBI (3). Injury-specific factors seem to play the largest prognostic role early on, whilst general factors such as resilience, access to social support, and degree of pre- and co-morbid psychological problems, play a greater role in long-term adjustment (4).

There are a limited number of well-designed TBI studies examining determinants of participation by the individuals with TBI, effective rehabilitation and community re-entry programs, and long-term outcomes. Rehabilitation studies from different countries are required to allow a better understanding of sociopolitical and cultural variation in patient needs and service delivery.

This e-book comprises 12 original research articles and two reviews from Australia, Canada, China, Denmark, France, Netherlands, Norway, and USA. The materials provide insights into the impact of TBI on physical, cognitive, emotional, behavioral and psychosocial functioning, participation in daily life activities and work, driving behavior, vocational rehabilitation, the role of peer support groups, financial compensation following TBI, and classification of health-related rehabilitation services.

The book opens with an original article by Wardlaw et al., which assessed the association of resilience, demographic, injury-related, cognitive, emotional, and family factors with participation following TBI. The study demonstrated that, across the full spectrum of injury severity, and persisting disabilities, resilience can impact on community reintegration many years post injury. Understanding the role of resilience can promote optimistic and hopeful treatment approaches.

The article by Shao et al. found that patients with mild TBI had reduced cortical thickness in the left entorhinal cortex while increased cortical thickness in the left precuneus cortex and right lateral occipital cortex. Female patients also had an increased cortical thickness in the left caudal anterior cingulate cortex compared to males. Increased cortical thickness was positively related to post-traumatic stress complaints in female patients. Sex differences in cortical thickness may be used as a neuroimaging phenotype for investigating clinical profiles of mild TBI.

In their review article, Polinder et al. discussed current evidence and controversies concerning the use of the terms post-concussion symptoms vs. syndrome, its diagnosis, etiology, prevalence, assessment, and treatment in both adults and children. The authors highlighted that post-concussion symptoms are dependent on complex interactions between somatic, psychological, and social factors, and that treatment is variable, and primarily directed at symptom relief, rather than at modifying underlying pathology.

An original article by Tibæk et al. investigated return to work in young persons (<30 years) with acquired brain injury, over a 10 years period. About one third had not achieved stable return to work and had much lower odds compared to controls for stable work attachment. No improvements in return to work were obtained after 2–5 years. Given the economic and social benefits of work, this result presents a major rehabilitation challenge.

Dornonville de la Cour et al. described a multidisciplinary, holistic and individually tailored vocational rehabilitation (VR) program for individuals with mild traumatic brain injury (mTBI). Both number of hours at work and RTW-status improved, with 97% having returned to work after VR. Shorter time since injury and male sex predicted a greater increase in working hours. The results suggest that individuals with mTBI may improve employment outcomes even years after injury with vocational rehabilitation support, and these preliminary findings demonstrate the need for further research into VR.

Winter et al. investigated potential mediation of variables influencing employment status following TBI in U.S. military veterans. Pain predicted employment status, but its effect was attenuated by physical health and functioning. Physical functioning effects were also attenuated when depressive symptoms were accounted for. This study illustrates the value of mediation analyses in yielding insights into predictors of employment status after TBI, particularly in tertiary prevention of poor TBI outcomes.

Howe et al. examined trajectories of employment probability up to 10 years following moderate-to-severe TBI, and found that overall probability of employment remained relatively stable at ~50% between 1-, 2-, 5-, and 10 years. Male gender, individuals in a partnered relationship at the time of injury, those employed at the time of injury, in a white-collar profession, and participants with higher acute injury severity had higher employment probability trajectories across the follow-up times. Regular follow-up is recommended for patients at risk of long-term unemployment.

Forslund et al. described longitudinal trajectories of overall disability assessed with the Glasgow Outcome Scale Extended (GOSE) in the first 10 years after moderate-to-severe TBI. They found that 37% of survivors experienced deterioration in disability levels between 5- and 10-year follow-ups, supporting the concept of TBI as a chronic health condition. Younger age, pre-injury employment, male gender, white collar occupation and shorter duration of post-traumatic amnesia are prognostic of better long-term global outcomes. Intensive and tailored rehabilitation may be required to counteract negative developments in disability levels.

The original article by Soendergaard et al. investigated neurobehavioral difficulties following severe brain injury as reported by both the survivor and their close family member using the St Andrew's-Swansea Neurobehavioral Outcome Scale (SASNOS). One fourth of the patients reported problems in Interpersonal Behavior and Cognition. Significant associations were found between proxies' reports and time since injury, cohabitant status, and the patient's score on the GOSE, and differences were seen between patient and proxy ratings. The problems reported by survivors and their proxies can affect the survivor's ability to reintegrate and participate in activities of daily living, emphasizing need for systematic assessment and tailored intervention.

In their prospective 8-years outcome study from a Parisian cohort with severe TBI, Ruet et al. showed that cognitive complaints were common, with ~70% reporting impaired mental speed, concentration and memory. Comparably, 30–40% had somatic complaints, and about one fourth experienced emotional distress. About half were in productive work. Only 20% showed good recovery on the GOSE, indicating that persisting impairments interfere with social integration and participation 8 years after injury.

In the same Parisian cohort, Bayen et al. investigated the relationship between compensation amounts and injury outcomes in litigants at 4 and/or 8 years after injury. Compensation amounts were positively associated with severity of disability and cognitive impairment, and with care time provided by caregivers. No association was seen with gender, age, education, motor/balance impairment, return to work status, mood or caregiver's subjective burden.

McKerral et al. explored driving behavior after TBI in individuals whose drivers' licenses had been suspended and reinstated following rehabilitation compared with individuals with TBI who did not have any suspension, and with non-injured controls. The study documents that the demerit points in official driving records increase significantly after a TBI. The suspended individuals reported lower level of verbal aggression and driving related errors compared to controls. Serious traffic accidents were higher post-injury in the suspended groups, and serious accidents increased despite the individuals' self-evaluation of being safe drivers. This underscores the need for careful examination of driving ability after TBI and may suggest need for an even stricter practice.

The original article by Bakmann et al. concerns a particularly vulnerable group of patients with acquired brain injury, namely adolescents and young adults. In addition to neurological and cognitive impairment, they are faced with issues concerning education, job, family, and social life. The paper emphasized how young survivors of brain injury benefit socially and psychologically from meeting like-minded peers in a peer support group, and how this may promote psychosocial recovery in adolescents and young adults with ABI.

The last review article by Røe et al. applied the International Classification System for Service Organization in Health-related Rehabilitation (ICSO-R) in a review of randomized intervention trials targeting moderate to severe traumatic brain injured persons in the post-acute phase. Few studies targeted these factors directly in their designs and analysis. However, service provision and delivery often varied between intervention arms in the studies, which could confound outcome evaluations. More standardized reporting of key factors of service provision and delivery in rehabilitation trials is needed.

## Author Contributions

All authors listed have made a substantial, direct and intellectual contribution to the work, and approved it for publication.

### Conflict of Interest

The authors declare that the research was conducted in the absence of any commercial or financial relationships that could be construed as a potential conflict of interest.
